# Ammonia in the Household

**Published:** 1891-11

**Authors:** 


### AMMONIA IN THE HOUSEHOLD.

If you wish to clean and brighten your carpets after they have been beaten and put down, wipe with a cloth wrung from water, to which a little ammonia has been added. A tablespoonful of ammonia in a gallon of water will often restore colors in carpets; it will also remove whitewash stains from them. In fact, the housekeeper has no better help than her bottle of ammonia. A few drops in a cupful of warm water, carefully applied with a soft rag, will clean paintings and chromos. Again, it will clean brass.

